# DPEA-Net: a clinically-oriented lightweight 3D CNN for glioma segmentation in multiparametric MRI

**DOI:** 10.3389/fneur.2026.1832698

**Published:** 2026-05-25

**Authors:** Caijian Hua, Xuerong Jing, Liuying Li, Xia Zhou

**Affiliations:** 1 School of Computer Science, Sichuan University of Science & Engineering, Zigong, China; 2 Traditional Chinese Medicine Department, Zigong First People's Hospital, Zigong, China

**Keywords:** computational efficiency, cross-dimensional attention, dynamic hierarchical convolution, glioma subregion segmentation, lightweight 3D CNN, multiparametric MRI analysis, neuroimaging pipeline

## Abstract

**Objective:**

Accurate segmentation of glioma subregions, whole tumor (WT), tumor core (TC), and enhancing tumor (ET), from multiparametric MRI is essential for radiotherapy planning and longitudinal assessment.

**Methods:**

We propose DPEA-Net, a lightweight 3D architecture that addresses these challenges through two novel components. First, the Dynamic Hierarchically Decoupled Convolution (DHDC) unit reduces parameters by 99% compared to 3D U-Net while enabling adaptive multi-scale feature extraction to handle tumor heterogeneity. Second, the Cross-Dimensional Region-Specific Enhancement Attention (CDRSEA) module explicitly models 3D spatial relationships to refine ambiguous tumor boundaries.

**Results:**

On the BraTS 2019 and 2020 validation sets, DPEA-Net achieves mean Dice scores of 90.43%/89.96% (WT), 85.56%/86.52% (TC), and 81.89%/80.31% (ET) with a computational footprint of only 17.48 Giga Floating-point Operations (GFLOPs), enabling sub-2-second inference on standard clinical hardware. A 1.5-fold TC weighting strategy further enhances segmentation of the clinically critical tumor core.

**Conclusion:**

DPEA-Net provides an accurate and computationally efficient tool for automated glioma subregion delineation, supporting practical integration into neuro-oncological workflows.

## 1 Introduction

Glioma is one of the most common and aggressive primary brain tumors, associated with high morbidity and mortality (1). Accurate and automated segmentation of its subregions, namely the WT, TC, and ET, from multiparametric MRI is a fundamental preprocessing step in quantitative neuroimaging. These subregions are defined as follows: ET corresponds to the actively proliferating tumor region with contrast enhancement on T1-weighted post-contrast images; TC comprises the ET together with the necrotic and non-enhancing tumor components; and WT encompasses the entire visible abnormality, including the TC and the surrounding peritumoral edema (2).

Multiparametric MRI provides complementary tissue contrast information essential for delineating these subregions. Specifically, T1-weighted imaging (T1) offers detailed anatomical structure; T1-weighted contrast-enhanced imaging (T1ce) highlights regions of blood-brain barrier disruption, which is essential for identifying the ET; T2-weighted imaging (T2) reveals both tumor and edema; and fluid-attenuated inversion recovery (FLAIR) suppresses cerebrospinal fluid signal to clearly delineate peritumoral edema, which is critical for defining the WT boundary (3, 4). The complementary nature of these sequences enables comprehensive characterization of glioma heterogeneity.

Accurate segmentation enables critical downstream tasks such as volumetric assessment, radiomic feature extraction, and longitudinal monitoring (3, 5). Manual segmentation is time-consuming, subjective, and difficult to scale, creating a bottleneck in large-scale research and clinical studies (6).

In neuro-oncological practice, precise subregion segmentation directly impacts surgical planning, radiotherapy dose prescription, and response assessment (2, 7). The TC and ET are particularly critical, as they represent the most aggressive components guiding biopsy targets and radiation boost volumes (8). However, manual delineation is subject to high inter-rater variability, posing challenges for reproducible quantitative analysis in multicenter trials and routine care. AI-driven image analysis has demonstrated significant potential in improving diagnostic accuracy and treatment planning for various brain tumors, including brain metastases (9), and similar benefits are expected for glioma subregion segmentation. More broadly, artificial intelligence techniques have been successfully applied across diverse medical domains, including explainable AI for early Alzheimer's diagnosis using multimodal data (10), deep learning-based arrhythmia classification (11), and mental health assessment through social media text analysis (12), underscoring the versatility and clinical promise of these computational approaches. Given these clinical demands, there is an urgent need for accurate, efficient, and deployable automated segmentation tools to support robust and reproducible neuroimaging workflows.

Convolutional neural networks (CNNs) have revolutionized brain tumor research, enabling automated segmentation, classification, and even molecular marker prediction from medical images (13). Recent surveys have systematically reviewed deep learning-based brain tumor segmentation models, categorizing them into CNN-based, vision transformer-based, and hybrid architectures (14). With the rapid advancement of artificial intelligence, deep learning-based methods have become the de facto standard for automated brain tumor segmentation, achieving promising results (15–17). However, their adoption is limited by a trade-off between accuracy and computational cost (18, 19). This inefficiency limits their practical use in resource-constrained research environments or when processing large-scale datasets. For instance, while models such as 3D U-Net provide a strong baseline, their high parameter counts make them prohibitive for many real-world applications (20, 21). Other approaches designed for efficiency, such as HDC-Net (22) or TDPC-Net (23), often compromise on adaptively capturing the heterogeneous scales of gliomas or modeling full 3D contextual information, leading to suboptimal segmentation of critical subregions (8). Recent advances in lightweight architecture design, including multiscale attention mechanisms (8, 24), feature fusion strategies (25), and cascaded efficient 3D networks (21), further illustrate the ongoing pursuit of computational efficiency without sacrificing accuracy. Thus, developing lightweight yet accurate segmentation tools remains a significant methodological challenge in neuroinformatics.

To address this gap, we present Dynamic Pathway-Enhanced Attention Network (DPEA-Net), a lightweight 3D convolutional network designed as an efficient computational tool for glioma segmentation. The model integrates two novel components: a Dynamic Hierarchically Decoupled Convolution (DHDC) unit and a Cross-Dimensional Region-Specific Enhancement Attention (CDRSEA) module. The DHDC unit enables adaptive multi-scale feature extraction with minimal parameters, directly tackling the challenge of tumor heterogeneity. The CDRSEA module explicitly models 3D spatial relationships to refine ambiguous boundaries, a persistent challenge in volumetric neuroimaging analysis. By achieving state-of-the-art accuracy on clinically relevant subregions (TC and ET) with minimal computational footprint, DPEA-Net has the potential to standardize glioma assessment and facilitate integration into neuro-oncological research pipelines. Together, these innovations yield a tool that combines state-of-the-art accuracy with exceptional efficiency, making it suitable for integration into diverse neuroimaging workflows.

The main contributions of this study are summarized as follows:

- We propose a DHDC unit that adaptively adjusts receptive fields to capture multi-scale tumor features with 99% fewer parameters than 3D U-Net, addressing glioma heterogeneity.
- The DHDC unit efficiently captures multi-scale tumor features via dynamic hierarchical decoupling and attention-based fusion, enhancing adaptability to heterogeneous tumors.
- The CDRSEA module addresses ambiguous tumor boundaries, improving subregion segmentation accuracy through cross-dimensional interaction and region-specific enhancement.
- Comprehensive validation on the BraTS 2019 and 2020 datasets demonstrates that DPEA-Net achieves Dice scores of 90.43%/89.96% (WT), 85.56%/86.52% (TC), and 81.89%/80.31% (ET) with only 17.48 GFLOPs, outperforming both mainstream and lightweight models.

## 2 Method

### 2.1 Dataset and preprocessing

We used the publicly available BraTS 2019 training dataset and BraTS 2020 training dataset for model training and validation (2, 20, 26). All BraTS datasets include Multiparametric MRI sequences: T1, T1ce, T2, and FLAIR. These sequences are widely used in neuroimaging studies for glioma analysis (22, 25, 27). Each case is accompanied by expert-annotated ground truth defining three glioma subregions: WT, TC, and ET.

Before training, we applied a series of preprocessing steps to standardize the data and enhance model generalization. First, intensity normalization was performed: the intensity of each MRI sequence was scaled to the range [0, 1] using min-max normalization to eliminate confounding effects from different scanning equipment and parameter settings. To balance computational efficiency and data diversity, we randomly cropped 128^3^ patches from the original 240 × 240 × 155 BraTS volumetric data during training, enhancing the model's generalization by sampling diverse spatial regions in each iteration.

During training, several online data augmentation techniques were applied to mitigate overfitting. Specifically, input volumes were randomly cropped from 240 × 240 × 155 to 128 × 128 × 128 voxels, randomly rotated within a range of ±10°, and subjected to random intensity variation with parameters (0.1, 0.1). Additionally, random flipping was performed along the 0-th axis.

During inference, the original images were first extended using padding technique (from 240 × 240 × 155 to 240 × 240 × 160) to ensure proper functioning of the network. Each MRI volume in the dataset has dimensions of 240 × 240 × 155 voxels, with an isotropic voxel spacing of 1 × 1 × 1mm^3^. Ground truth annotations were manually delineated by expert clinicians, who categorized the tumor regions into three subregions: WT, TC, and ET.

### 2.2 Overall architecture of DPEA-Net

**Figure 1:**
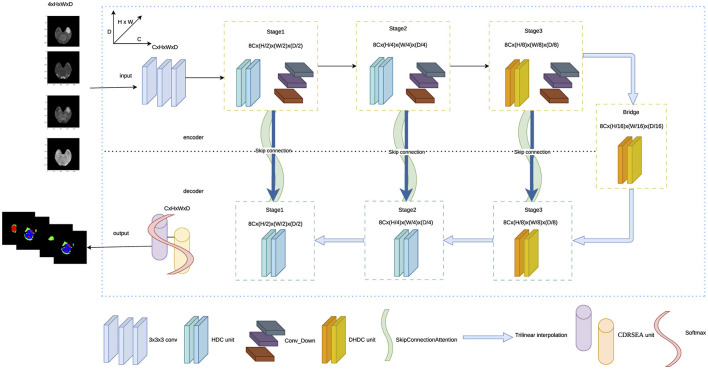
Encoder-decoder structure integrating DHDC for multi-scale feature extraction and CDRSEA for boundary refinement.

The detailed architecture of DPEA-Net, including the integration of the DHDC and CDRSEA modules, is illustrated in Figure 1. As a lightweight 3D CNN designed for precise glioma subregion segmentation (17, 27–29), DPEA-Net's core design goal is to balance deployability and segmentation accuracy. The network adopts an encoder-decoder structure: the encoder extracts multi-scale tumor features from Multiparametric MRI data, while the decoder reconstructs the spatial structure of glioma subregions to generate high-precision segmentation masks. To address key challenges such as tumor heterogeneity and boundary ambiguity, the two core modules are integrated into the encoder and decoder, respectively. This architecture is specifically designed to reduce computational overhead while maintaining high segmentation performance, enabling deployment on standard clinical workstations.

For a detailed overview of the network composition, Table 1 summarizes the architectural specifications of DPEA-Net, including layer types, kernel sizes, stride configurations, and channel dimensions at each stage of the encoder-decoder pathway. The complete model contains 24 operational layers and achieves a compact footprint of 0.17M parameters with a computational cost of 17.48 GFLOPs.

**Table 1:** Architectural specifications of DPEA-Net.

Stage	Layer ID	Layer type	Kernel size	Stride	In/out channels
Input	1	HDC spatial encoding	–	–	4 → 32
Stem	2	Conv3D + BN + ReLU	3 × 3 × 3	1	32 → 32
Encoder 1	3	HDC module	–	–	32 → 32
	4	Downsampling Conv	3 × 3 × 3	2	32 → 32
Encoder 2	5	HDC module	–	–	32 → 32
	6	Downsampling Conv	3 × 3 × 3	2	32 → 32
Encoder 3	7	DHDC module	–	–	32 → 32
	8	Downsampling Conv	3 × 3 × 3	2	32 → 32
Bridge	9	DHDC module	–	–	32 → 32
Decoder 3	10	Trilinear upsample	–	×2	32 → 32
	11	Skip connection	–	–	32+32 → 64
	12	CDRSEA	–	–	64 → 64
	13	DHDC module	–	–	64 → 32
Decoder 2	14	Trilinear upsample	–	×2	32 → 32
	15	Skip connection	–	–	32+32 → 64
	16	CDRSEA	–	–	64 → 64
	17	HDC module	–	–	64 → 32
Decoder 1	18	Trilinear upsample	–	×2	32 → 32
	19	Skip connection	–	–	32+32 → 64
	20	CDRSEA	–	–	64 → 64
	21	HDC module	–	–	64 → 32
Output head	22	Trilinear upsample	–	×2	32 → 32
	23	CDRSEA	–	–	32 → 32
	24	1 × 1 × 1 Conv + Softmax	1 × 1 × 1	1	32 → 4

### 2.3 DHDC unit

**Figure 2:**
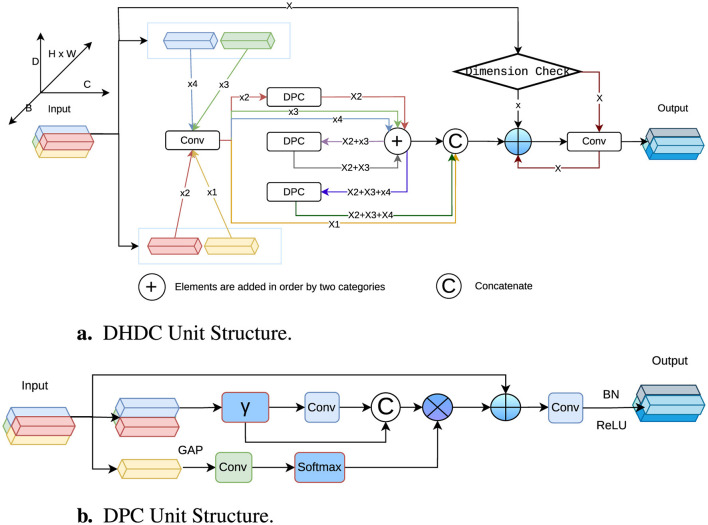
Core convolutional module structures of DPEA-Net. **(A)** Schematic of the dynamic hierarchically decoupled convolution (DHDC) unit **(B)** Schematic of the dynamic partial convolution (DPC) unit.

The structure and working mechanism of the DHDC unit are depicted in Figure 2. As the core feature extraction module of DPEA-Net, the DHDC unit is designed to efficiently capture multi-scale tumor features while reducing model parameters, addressing the computational bottleneck that limits the practical deployment of existing models. Its dynamic multi-branch design enables adaptive capture of features at varying scales, which is crucial for representing the heterogeneous tissue composition within gliomas, a key imaging characteristic for accurate subregion delineation. The key mathematical expressions for the DHDC unit are provided in Equations 1–3.

Specifically, the DHDC unit comprises three parallel convolution branches with different dilation rates, dynamically weighted and fused via an attention mechanism. The varying dilation rates allow the unit to capture both local details and global context. Gliomas exhibit significant heterogeneity, with the TC requiring fine-grained details and peritumoral edema requiring broader context. Fixed receptive fields cannot adapt to this variability. The proposed dynamic γ strategy uses global average pooling to generate data-driven attention weights for each dilation rate, enabling the model to automatically select the optimal receptive field based on local image characteristics. Meanwhile, the attention fusion mechanism adaptively adjusts the weight of each branch based on the input feature map, ensuring that the module focuses more on tumor-related regions while suppressing irrelevant background. Compared with static convolution modules, the DHDC unit achieves superior adaptability to the heterogeneous appearance of gliomas across different patients, addressing a key challenge in clinical radiological reading, while also reducing parameter counts and computational overhead. As highlighted in recent surveys (14), multi-scale feature extraction is crucial for capturing tumor heterogeneity, which our DHDC unit addresses through dynamic receptive field adjustment.

Formally, given an input feature tensor $X\in {\mathbb{R}}^{B\times C_{\text{in}}\times D\times H\times W}$, the DHDC unit first projects it to *X*_1_ = σ(BN(Conv_1 × 1 × 1_(*X*))) and splits *X*_1_ into four equal channel-wise branches $\left\{X_{1}^{1},X_{1}^{2},X_{1}^{3},X_{1}^{4}\right\}$. The latter three branches are processed by cascaded dynamic partial convolutions:


$$\begin{array}{l} \begin{array}{cc} X_{1}^{2^{\prime }} & =\text{DynamicPartialConv}\left(X_{1}^{2}\right),\\ X_{1}^{3^{\prime }} & =\text{DynamicPartialConv}\left(X_{1}^{3}+X_{1}^{2^{\prime }}\right),\\ X_{1}^{4^{\prime }} & =\text{DynamicPartialConv}\left(X_{1}^{4}+X_{1}^{3^{\prime }}\right), \end{array} \end{array}$$ (1)

where the dynamic partial convolution adaptively fuses multiple dilation branches via learned attention weights:


$$\begin{array}{l} \text{DynamicPartialConv}\left(Z\right)= \end{array}$$

$$\begin{array}{l} \sigma \left(\text{BN}\left({\text{Conv}}_{1\times 1\times 1}\left(\overset{3}{\underset{k=1}{\sum }}\alpha_{k}\cdot B_{k}\left(Z\right)+Z\right)\right)\right). \end{array}$$ (2)

Here *B*_*k*_(·) denotes the *k*-th dilated convolution branch (with dilation rates γ∈{2, 4, 8}) and α_*k*_ = Softmax(Conv_1 × 1 × 1_(GAP(*Z*))) are data-driven attention weights obtained via global average pooling (GAP). The final output is obtained by concatenating all branches and applying a residual connection:


$$\begin{array}{l} Y_{\text{out}}=\sigma \left(\text{BN}\left({\text{Conv}}_{1\times 1\times 1}\left(\hat{X}⊕\overset{4}{\underset{i=1}{⊕}}X_{1}^{i^{\prime }}\right)\right)\right), \end{array}$$ (3)

with $\hat{X}$ being an optional 1 × 1 × 1 projection when *C*_in_≠*C*_out_, and ⊕ denoting channel-wise concatenation. Further implementation details and the complete derivation are provided in the Supplementary material. The effectiveness of this formulation is validated through ablation experiments in Section 3.2.

### 2.4 CDRSEA module

**Figure 3:**
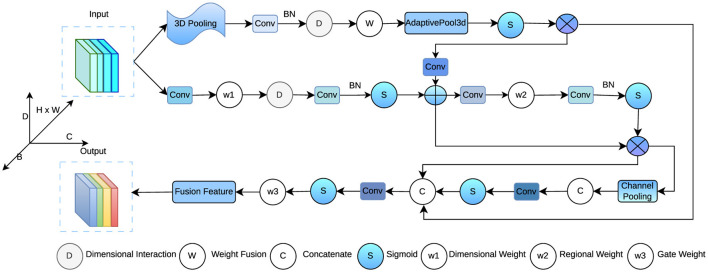
Cross-dimensional spatial interaction and region-specific attention for boundary enhancement.

The structural details and feature interaction process of the CDRSEA module are illustrated in Figure 3. Integrated into the decoder of DPEA-Net, this module focuses on resolving ambiguous tumor boundaries in Multiparametric MRI, a major challenge in volumetric glioma segmentation. The cross-dimensional interaction mechanism in CDRSEA is specifically designed to explicitly model the 3D contextual relationships that radiologists use when integrating information across axial, coronal, and sagittal views to resolve ambiguous boundaries. Radiologists typically review MRI in axial, coronal, and sagittal planes simultaneously to resolve ambiguous tumor boundaries. The CDRSEA module explicitly models this cross-dimensional interaction using grouped convolutions with kernels (3,3,1), (3,1,3), and (1,3,3), simulating the multi-planar reconstruction (MPR) process in clinical practice. In doing so, it mimics the MPR review process used by radiologists, thereby enhancing clinical interpretability. The key mathematical expressions for the CDRSEA module are provided in Equations 4–7.

The CDRSEA module consists of three key components: cross-dimensional feature interaction, residual spatial enhancement, and attention weighting. First, cross-dimensional feature interaction extracts and fuses features from axial, coronal, and sagittal dimensions, capturing the 3D spatial relationships between tumor subregions and surrounding tissues. This addresses the limitation of single-dimensional feature extraction, which often fails to capture the complete 3D structure of gliomas. Second, the residual spatial enhancement component improves feature representation of tumor boundaries through residual connections, helping preserve detailed boundary information critical for accurate analysis. Finally, the attention weighting mechanism highlights features of clinically critical subregions, further improving segmentation accuracy.

Formally, let *X*∈ℝ^*B*×*C*×*D*×*H*×*W*^ be the input feature map. The CDRSEA module first computes a global spatial calibration map $A_{\text{base}}$ via channel-wise average and max pooling, followed by cross-dimensional interaction:


$$\begin{array}{l} A_{\text{base}}=\sigma \left(A_{\text{global}}\left(X_{aHWD}\right)\right)+\sigma \left(M_{\text{global}}\left(X_{mHWD}\right)\right), \end{array}$$ (4)

where *X*_*aHWD*_ and *X*_*mHWD*_ denote the dimension-permuted tensors for average- and max-pooling branches, respectively. The calibrated features are then enhanced by cross-dimensional attention:


$$\begin{array}{l} X_{\text{cross}}=X_{\text{base\_atten}}+\underset{\left(d_{1},d_{2},d_{3}\right)\in \left\{\left(3,3,1\right),\left(3,1,3\right),\left(1,3,3\right)\right\}}{\sum } \end{array}$$

$$\begin{array}{l} \beta_{d_{1}d_{2}d_{3}}\cdot \text{GroupConv}\left(X_{\text{base\_atten}},\left(d_{1},d_{2},d_{3}\right)\right), \end{array}$$ (5)

where $X_{\text{base\_atten}}={\text{Conv}}_{1\times 1\times 1}\left(X⊙A_{\text{base}}\right)$, ⊙ denotes element-wise multiplication, and the attention weights β are generated via global pooling and a 1 × 1 × 1 convolution. To further emphasize clinically critical subregions, region-specific gating is applied:


$$\begin{array}{l} X_{\text{region}}=X_{\text{cross}}⊙\left(1+\text{RegionAttn}\left(X_{\text{cross}}\right)\right), \end{array}$$ (6)

with RegionAttn(·) producing gating coefficients [*g*_WT_, *g*_TC_, *g*_ET_] through three independent 1 × 1 × 1 convolutions; notably, *g*_TC_ is scaled by a factor of 1.5 to address class imbalance. Finally, an adaptive gated fusion combines the calibrated and region-enhanced features:


$$\begin{array}{l} Y_{\text{out}}=G⊙\left(X⊙A_{\text{base}}\right)+\left(1-G\right)⊙X_{\text{region}}, \end{array}$$ (7)

where $G=\sigma \left(\text{Conv}\left(\text{Concat}\left(X⊙A_{\text{base}},X_{\text{region}}\right)\right)\right)$ is a learnable fusion gate. By integrating cross-dimensional context and region-specific enhancement, the CDRSEA module effectively refines ambiguous tumor boundaries. Additional architectural details and ablation results are available in the Supplementary material.

### 2.5 Key hyperparameter settings

To ensure optimal performance and deployment efficiency, we conducted systematic sensitivity analyses on two critical hyperparameters: the receptive field adjustment factor γ in the DHDC unit and the TC weight scaling factor in the loss function. These analyses were designed to determine the optimal configuration balancing segmentation accuracy, computational complexity, and clinical relevance.

For the DHDC unit, we evaluated both fixed γ values (2, 4, 8) and a dynamic γ adjustment strategy. The dynamic strategy generates data-driven attention weights via global average pooling, enabling adaptive receptive field selection based on local image characteristics.

For the loss function, we investigated TC weight scaling factors ranging from 1.0 to 2.0 to address the inherent class imbalance caused by the small volume of the TC region relative to the WT and surrounding normal tissue.

Based on comprehensive empirical validation (detailed in Section 3.4), the optimal hyperparameter combination was determined as follows: dynamic receptive field adjustment for the DHDC unit and a 1.5-fold scaling factor for the TC loss weight. This configuration forms a Pareto-optimal solution underpinning DPEA-Net's state-of-the-art performance.

### 2.6 Training and evaluation metrics

DPEA-Net was implemented using the PyTorch framework and trained on a standard clinical workstation equipped with an NVIDIA Tesla P100 GPU, reflecting realistic deployment conditions. To address class imbalance in glioma subregion segmentation, we primarily used Generalized Dice Loss (GDL); mixed loss variants combining GDL with Focal Loss were also explored. Following standard practices in neuroimaging segmentation benchmarks, we used five evaluation metrics: DSC, Sensitivity (Sen), Specificity (Spe), Precision (Pre), and HD_95_. DSC was selected as the primary metric, measuring the overlap between predicted segmentation masks and expert-annotated ground truth. Sensitivity evaluates the model's ability to correctly identify tumor regions, while precision assesses its capability to avoid false positives, both of which are essential for reliable quantitative analysis. Specificity quantifies the model's ability to correctly identify normal brain tissue, complementing sensitivity and precision to provide a comprehensive reliability assessment (30). HD_95_ measures the maximum distance between predicted and ground truth boundaries and reflects boundary delineation accuracy; lower values indicate better alignment with expert annotations. These metrics are used throughout the Results section, and their mathematical formulations are provided in the Supplementary material. The GDL is defined as Equation 8:


$$\begin{array}{l} L_{\text{GDL}}=1-\frac{2\overset{K}{\underset{c=1}{\sum }}w_{c}\overset{N}{\underset{i=1}{\sum }}p_{c,i}g_{c,i}}{\overset{K}{\underset{c=1}{\sum }}w_{c}\overset{N}{\underset{i=1}{\sum }}\left(p_{c,i}+g_{c,i}\right)} \end{array}$$ (8)

where *K* = 4 (background, WT, TC, ET); $w_{c}=1/{\left(\overset{N}{\underset{i=1}{\sum }}g_{c,i}\right)}^{2}$ is the class weight inversely proportional to region volume; *p*_*c, i*_ is the predicted probability of voxel *i* for class *c*; and *g*_*c, i*_ is the binarized ground truth value (0/1) for voxel *i* of class *c*.

The Adam optimizer was employed with an initial learning rate of, which was dynamically adjusted using a power decay strategy to improve training stability and convergence. Early stopping with a patience of 50 epochs was applied to terminate training, where the validation loss was monitored to determine convergence. Training was terminated after a maximum of 500 epochs, and the best model checkpoint was selected based on the highest average validation Dice score across all tumor subregions. The training and validation loss curves, along with the evolution of Dice scores across epochs, are provided in the Supplementary Figure S1, demonstrating stable convergence without overfitting.

The Adam optimizer was employed with an initial learning rate of 1 × 10^−4^, which was dynamically adjusted using a power decay strategy to improve training stability and convergence. Early stopping with a patience of 50 epochs was applied to terminate training by monitoring the loss. Training was terminated after a maximum of 500 epochs, and the best model checkpoint was selected based on the lowest loss. The loss curves, along with the evolution of Dice scores across epochs, are provided in the Supplementary Figure S1, demonstrating stable convergence without overfitting.

## 3 Results

### 3.1 Quantitative comparison with state-of-the-art methods

To comprehensively evaluate DPEA-Net, we conducted extensive experiments on the BraTS 2019 and 2020 official validation sets, comparing it against mainstream heavyweight models and state-of-the-art lightweight models. The quantitative results are summarized in Tables 2–4, including DSC, Sen, Spe, and HD_95_ for the three glioma subregions (WT, TC, and ET) defined earlier.

**Table 2:** Quantitative segmentation performance of DPEA-Net on the BraTS 2019 and 2020 validation datasets.

Dataset	DSC (%)↑			Sensitivity (%)↑			Specificity (%)↑			Precision (%)↑		
	WT	TC	ET	WT	TC	ET	WT	TC	ET	WT	TC	ET
BraTS2019	90.43	85.56	81.89	90.39	82.87	77.17	99.92	99.96	99.97	91.23	91.51	77.34
BraTS2020	89.96	86.52	80.31	88.78	86.99	77.74	99.88	99.93	99.95	92.19	88.78	76.03

WT, whole tumor; TC, tumor core; ET, enhancing tumor; DSC, dice similarity coefficient. Arrows indicate whether higher (↑) or lower (↓) values represent better performance.

**Table 3:** Comparative segmentation performance of DPEA-Net and state-of-the-art models on the BraTS 2019 validation dataset.

Model	M (M)↓	G (GFLOPs)↓	DSC (%) ↑			HD_95_ ↓		
			WT	TC	ET	WT	TC	ET
3D-UNet (20)	16.90	775.02	85.06	79.97	73.41	15.32	15.95	14.03
3D-VNet (31)	47.37	319.46	85.89	80.33	75.13	8.73	9.94	10.52
HDC-Net (22)	0.29	25.82	87.66	81.27	78.69	5.73	8.35	4.54
DMF-Net (32)	3.88	27.87	88.93	83.79	76.36	5.36	6.89	4.91
ADHDC-Net (8)	0.53	27.28	89.29	83.13	78.45	6.06	7.12	3.99
**DPEA-Net**	**0.17**	**17.48**	**90.43**	**85.56**	**81.89**	**4.87**	**5.52**	**3.08**

M, number of parameters (million); G, giga floating-point operations (GFLOPs); DSC, Dice similarity coefficient; HD_95_, 95th percentile Hausdorff distance; WT, whole tumor; TC, tumor core; ET, enhancing tumor. Arrows indicate whether higher (↑) or lower (↓) values represent better performance. Bold values denote the best performance for each metric among all settings.

**Table 4:** Comparative segmentation performance of DPEA-Net and state-of-the-art models on the BraTS 2020 validation dataset.

Model	M (M)↓	G (GFLOPs)↓	DSC (%) ↑			HD_95_ ↓		
			WT	TC	ET	WT	TC	ET
Swin-Unet (33)	27.15	250.88	89.82	77.47	79.39	10.32	15.39	7.88
3D-ESPNet (34)	3.21	74.36	89.05	84.92	76.47	6.55	9.84	**6.98**
HDC-Net (22)	0.29	25.82	89.59	84.35	78.34	6.64	9.77	11.07
DMF-Net (32)	3.88	27.87	90.06	83.77	77.81	5.32	10.49	15.39
TDPC-Net (23)	0.19	17.96	**90.19**	84.91	78.95	4.88	9.59	12.33
**DPEA-Net**	**0.17**	**17.48**	89.96	**86.52**	**80.31**	**4.22**	**8.39**	10.91

M, number of parameters (million); G, giga floating-point operations (GFLOPs); DSC, Dice similarity coefficient; HD_95_, 95th percentile Hausdorff distance; WT, whole tumor; TC, tumor core; ET, enhancing tumor. Arrows indicate whether higher (↑) or lower (↓) values represent better performance. Bold values denote the best performance for each metric among all settings.

As shown in Tables 3, 4, DPEA-Net achieved superior performance across all evaluation metrics while maintaining high computational efficiency and a lightweight profile. Specifically, on the BraTS 2019 validation set, DPEA-Net achieved Dice scores of 90.43% (WT), 85.56% (TC), and 81.89% (ET); on the BraTS 2020 validation set, the corresponding scores were 89.96% (WT), 86.52% (TC), and 80.31% (ET). Notably, the TC Dice scores represent critical improvements over baseline models. Additionally, the sub-millimeter reduction in HD_95_ for ET indicates that our model is particularly effective at refining boundaries of invasive tumor regions, crucial for precise radiotherapy planning.

In terms of computational efficiency, DPEA-Net outperformed all compared models, achieving a 99% parameter reduction compared with 3D U-Net and a 10%–35% reduction in GFLOPs compared with lightweight models such as HDC-Net and TDPC-Net. This lightweight advantage ensures deployability on standard clinical workstations, addressing a key barrier to adoption in research pipelines.

To further validate the statistical significance of these improvements, we conducted paired *t*-tests comparing Dice and HD_95_ scores against DFM-Net, HDC-Net, and 3D U-Net on the BraTS 2019 validation set. DPEA-Net demonstrated statistically significant improvements (*p* < 0.05) across all three subregions for all baseline comparisons. Notably, the most pronounced differences were observed in ET Dice scores (all *p* < 0.001) and TC HD_95_ values (*p* < 0.01). The complete statistical results, including mean values, standard deviations, *t*-statistics, and exact *p*-values, are provided in Supplementary Table S1.

### 3.2 Ablation study of core modules

To systematically evaluate the individual and synergistic contributions of our proposed components (trilinear interpolation, TI; DHDC unit; and CDRSEA, module), we conducted a progressive ablation study. We evaluated six configurations on the BraTS 2019 validation set, starting from a baseline HDC-Net: (1) Baseline (HDC-Net), (2) Baseline + TI, (3) Baseline + DHDC, (4) Baseline + TI + DHDC, (5) Baseline + CDRSEA, and (6) DPEA-Net (all components integrated). The results are summarized in Table 5. The baseline model was a lightweight 3D encoder-decoder network without the DHDC and CDRSEA modules.

**Table 5:** Ablation study results of DPEA-Net's core components on the BraTS 2019 validation dataset.

Model	M (M)↓	G (GFLOPs)↓	DSC (%) ↑			HD_95_ ↓		
			WT	TC	ET	WT	TC	ET
Baseline	0.29	25.82	87.66	81.27	78.28	5.73	8.35	4.54
Baseline+TI	0.21	19.37	88.38	83.19	77.79	4.82	7.36	4.09
Baseline+DHDC	0.27	24.69	88.98	83.74	78.66	5.89	6.25	3.88
Baseline+TI+DHDC	0.19	**17.47**	89.93	83.79	78.94	6.36	6.54	3.01
Baseline+CDRSEA	0.23	26.58	89.69	84.54	79.05	5.97	6.22	3.85
**DPEA-Net**	**0.17**	17.48	**90.43**	**85.56**	**81.89**	**4.87**	**5.52**	**3.08**

M, number of parameters (million); G, giga floating-point operations (GFLOPs); DSC, Dice similarity coefficient; HD_95_, 95th percentile Hausdorff distance; WT, whole tumor; TC, tumor core; ET, enhancing tumor. Arrows indicate whether higher (↑) or lower (↓) values represent better performance. Bold values denote the best performance for each metric among all settings.

Compared with the baseline, the model with only the TI module (Baseline+TI) achieved a 0.72% improvement in WT DSC, a 1.92% improvement in TC DSC, and a slight decrease of 0.49% in ET DSC. This indicates that the trilinear interpolation module effectively enhances the consistency of cross-slice features, thereby improving the segmentation accuracy of the WT and TC. The slight decrease in ET DSC may be due to the relatively small volume of the enhancing tumor, where excessive interpolation may introduce minor noise.

Compared with the baseline, the model with only the DHDC module (Baseline+DHDC) achieved a 1.32% improvement in WT DSC, a 2.47% improvement in TC DSC, confirming that the dynamic multi-scale feature extraction capability of the DHDC module effectively addresses the heterogeneity of glioma tissues. This aligns with the hierarchical feature extraction principle of CNNs (13), where shallow layers capture edges and deep layers encode semantics. In contrast, the model with only the CDRSEA module (Baseline+CDRSEA) achieved a 2.03% improvement in WT DSC, a 3.27% improvement in TC DSC, and a 0.77% improvement in ET DSC, demonstrating that the cross-dimensional attention mechanism of the CDRSEA module effectively resolves tumor boundary ambiguity.

When both DHDC and CDRSEA modules were integrated along with the TI module to form DPEA-Net, the highest DSC scores were achieved, indicating that the DHDC, CDRSEA, and TI modules complement each other and synergistically enhance the model's segmentation performance. Furthermore, the combination of TI with DHDC (Baseline+TI+DHDC) further improved the WT DSC to 89.93%, which is 0.95% higher than that of Baseline+DHDC, confirming the synergistic effect of TI in enhancing feature consistency and DHDC in multi-scale feature extraction. Notably, Baseline + TI + DHDC also achieved the lowest GFLOPs (17.47 G) among all ablation configurations, highlighting the efficiency advantage of the combined modules.

### 3.3 Qualitative visualization of segmentation results

Qualitative visualization provides evidence of the model's ability to handle challenging segmentation cases, such as ambiguous tumor boundaries, extensive peritumoral edema, and irregular morphology, factors that complicate automated analysis in volumetric neuroimaging.

**Figure 4:**
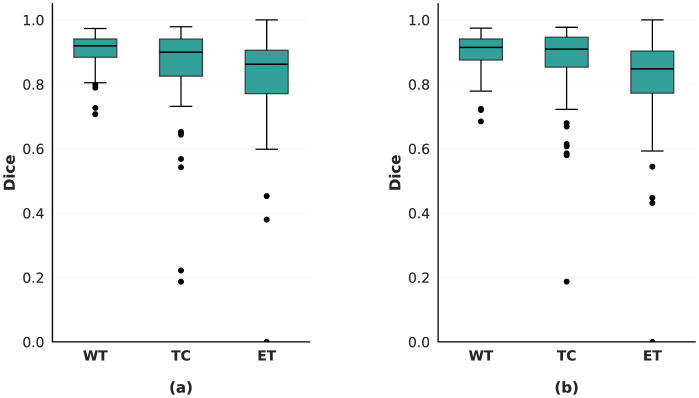
Box plot of dice similarity coefficient (DSC) scores of DPEA-Net for glioma subregion segmentation on the BraTS 2019 **(A)** and BraTS 2020 **(B)** validation datasets.

Figure 4 displays the distribution of DSC scores for DPEA-Net across the three glioma subregions on the BraTS 2019 and 2020 official validation sets. The boxplot is constructed in line with standard academic visualization specifications: the box represents the interquartile range (IQR) between the 25th and 75th percentiles, the horizontal line inside the box denotes the median DSC score, the whiskers extend to the minimum and maximum values within 1.5 × IQR, and outliers are marked as individual points. As illustrated in the boxplot, DPEA-Net exhibits high median DSC scores and narrow IQR across all three subregions on both datasets, indicating superior segmentation accuracy and excellent performance stability. Specifically, for the TC subregion, DPEA-Net's median DSC is 85.56% on BraTS 2019 and 86.52% on BraTS 2020, with a narrow IQR, demonstrating its robust adaptability to the heterogeneous appearance of gliomas across different patients and datasets. For the ET subregion, the most challenging to segment, DPEA-Net's median DSC is 81.89% on BraTS 2019 and 80.31% on BraTS 2020, with fewer outliers, which indicates consistent performance even for cases with ambiguous boundaries or complex tumor morphology. This boxplot visualization further confirms the quantitative superiority and stability of DPEA-Net, as detailed earlier, and provides intuitive evidence of its reliability, which is critical for practical deployment in research settings.

**Figure 5:**
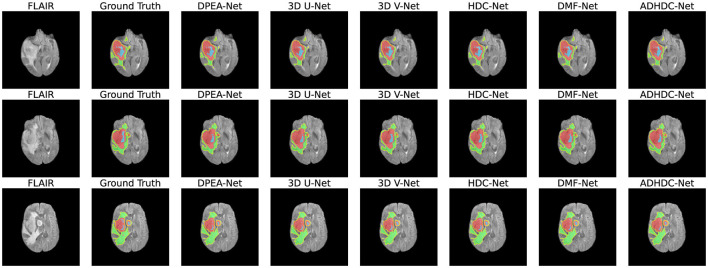
Qualitative visualization of segmentation results for a challenging glioma case with extensive peritumoral vasogenic edema on multiparametric MRI.

Figure 5 presents the 2D multi-planar reconstruction (MPR) views of a challenging case with extensive peritumoral vasogenic edema and an ill-defined tumor core, which is a typical clinical scenario that poses significant challenges for radiological segmentation. Consistent with clinical MRI reading workflows, the figure includes five distinct components, beginning with Multiparametric MRI input comprising FLAIR and T1ce sequences: FLAIR is the most sensitive sequence for detecting peritumoral edema and tumor infiltration, while T1ce is critical for identifying ET and distinguishing it from normal enhancing tissues, serving as two core sequences for glioma diagnosis and segmentation in neuroimaging analysis. Another component is the expert-annotated ground truth (gold standard), with WT in red and ET in yellow, consistent with clinical glioma subregion segmentation standards. The remaining three components display segmentation results from different models, including 3D U-Net, HDC-Net, and DPEA-Net. As observed in clinical image interpretation, 3D U-Net over-segments the peritumoral edema region into normal brain parenchyma, leading to overestimation of WT extent, which is a common error in clinical auxiliary segmentation that may misguide surgical planning and result in unnecessary normal tissue resection. Similarly, HDC-Net fails to capture the full extent of the enhancing tumor core and exhibits blurred boundaries between TC and surrounding edema, which is prone to missed diagnosis of tumor core regions and may increase the risk of tumor recurrence due to incomplete tumor resection. In contrast, DPEA-Net's segmentation aligns closely with the expert-annotated ground truth, demonstrating superior capability in distinguishing tumor infiltration from vasogenic edema and preserving the structure of small core regions, thereby effectively addressing a common challenge in clinical radiological readings and improving the reliability of automated segmentation for challenging cases in neuroimaging analysis.

### 3.4 Key hyperparameter analysis

Systematic ablation experiments and hyperparameter sensitivity analyses were conducted on the BraTS 2019 validation set to validate the rationality of DPEA-Net's core architectural designs and parameter configurations, with detailed results presented in Tables 6, 7. For the DHDC unit, fixed γ values (2, 4, 8) yielded consistent model complexity, with γ = 4 attaining the optimal baseline performance. In contrast, the dynamic γ strategy delivered a substantial performance boost with only marginal computational overhead, elevating TC DSC to 85.56% and ET DSC to 81.89%, while also achieving the minimum TC HD_95_ of 5.52 mm, this result directly demonstrates the strategy's superior adaptability to the heterogeneous spatial characteristics of gliomas, a key challenge in volumetric tumor segmentation, as lower HD_95_ values indicate more precise boundary delineation for clinical applications.

**Table 6:** Performance comparison of different receptive field adjustment factor (γ) settings in the DHDC unit on the BraTS 2019 validation dataset.

Model	M (M)↓	G (GFLOPs)↓	DSC (%) ↑			HD_95_ ↓		
			WT	TC	ET	WT	TC	ET
γ = 2	0.16	17.29	90.12	83.81	78.50	5.47	6.13	**2.91**
γ = 4	0.16	17.25	90.42	84.06	79.07	**4.78**	5.61	3.07
γ = 8	0.16	**17.21**	89.98	83.90	78.23	5.79	5.97	3.53
**Dynamic** **γ**	**0.17**	17.48	**90.43**	**85.56**	**81.89**	4.87	**5.52**	3.08

M, number of parameters (million); G, giga floating-point operations (GFLOPs); DSC, Dice similarity coefficient; HD_95_, 95th percentile Hausdorff distance; WT, whole tumor; TC, tumor core; ET, enhancing tumor. Arrows indicate whether higher (↑) or lower (↓) values represent better performance. Bold values denote the best performance for each metric among all settings.

**Table 7:** Sensitivity analysis of the TC weight scaling factor in the loss function on the BraTS 2019 validation dataset.

Setting	DSC (%)↑				TC sensitivity↑	HD_95_ (TC)↓
	WT	TC	ET	Average		
1.0	89.20	82.10	80.35	83.88	78.23	6.21
1.25	89.18	82.89	80.33	84.13	80.12	5.98
**1.5**	**90.43**	**85.56**	**81.89**	**85.96**	**82.87**	**5.52**
1.75	89.02	83.59	80.11	84.24	82.31	5.76
2.0	88.85	83.32	79.87	84.01	81.98	5.83

DSC, Dice similarity coefficient; HD_95_, 95th percentile Hausdorff distance; WT, whole tumor; TC, tumor core; ET, enhancing tumor. Arrows indicate whether higher (↑) or lower (↓) values represent better performance. Bold values denote the best performance for each metric among all settings.

Building on these module-level optimizations, we further analyzed the TC weight factor in the loss function to address class imbalance in glioma subregion segmentation. A 1.5-fold scaling of the default TC weight maximized both the clinical utility and overall segmentation accuracy of the model: this setting achieved the highest TC DSC (85.56%) and TC sensitivity (82.87%), alongside the maximum average DSC of 85.96%, while maintaining robust segmentation performance for the WT and ET subregions and the lowest TC HD_95_ (5.52 mm) across all tested configurations. Notably, weight factors exceeding 1.5 resulted in over-optimization on the TC region and a subsequent decline in segmentation accuracy for other subregions, whereas lower weight factors failed to effectively alleviate the inherent class imbalance caused by the small volume of the TC region relative to the WT and surrounding normal tissue. Collectively, the hyperparameter combination of the dynamic γ strategy for the DHDC unit and 1.5-fold TC weight scaling was validated to achieve a Pareto optimal balance among segmentation precision, computational efficiency and clinical relevance. This optimized parameter set not only underpins the superior performance of DPEA-Net observed in quantitative and qualitative evaluations, but also ensures the model's practical applicability for Multiparametric MRI-based glioma subregion segmentation in resource-constrained clinical and research settings.

## 4 Discussion

DPEA-Net achieves state-of-the-art glioma segmentation on BraTS with two-order-of-magnitude higher efficiency than conventional 3D CNNs. This efficiency–accuracy balance addresses a key barrier in neuroimaging: the high resource demands of existing models. Supported by comprehensive experimental results and systematic ablation analyses, we now discuss the methodological implications of our design and the practical utility of DPEA-Net as a neuroimaging analysis tool.

### 4.1 Methodological contributions to neuroimaging analysis

Accurate segmentation of glioma subregions is not merely a technical challenge but a clinical necessity, directly influencing therapeutic decisions in neuro-oncology, including surgical planning, radiotherapy dose prescription, and response assessment according to established criteria such as Response Assessment in Neuro-Oncology (RANO) (2). The ability to reliably delineate the TC and ET is particularly critical, as these regions represent the most aggressive components guiding biopsy targets and radiation boost volumes (8). However, achieving consistent and reproducible segmentation across diverse patient populations and imaging protocols remains a significant barrier to clinical translation. As noted in brain metastasis research (9), AI-driven image analysis has the potential to standardize assessment and reduce inter-observer variability, goals that DPEA-Net aims to achieve in glioma segmentation.

The DHDC unit addresses a fundamental clinical challenge: the marked heterogeneity of gliomas across patients, which complicates consistent radiological interpretation. By adaptively adjusting receptive fields based on local image context, it captures multi-scale tumor features, leading to more robust and reproducible segmentation across diverse patient populations, a prerequisite for reliable longitudinal and multi-center studies. As validated by our ablation experiments, the dynamic γ adjustment strategy outperforms fixed configurations, demonstrating that adaptive receptive field optimization is essential for handling the anatomical variability encountered in routine clinical practice.

The CDRSEA module explicitly models cross-dimensional spatial interactions, mirroring the multi-planar reconstruction (MPR) review process routinely used by radiologists to resolve ambiguous boundaries. This design is particularly beneficial for delineating the TC and ET, the primary targets for radiation dose escalation. By refining boundary precision, it directly supports accurate target volume definition in radiotherapy planning, potentially reducing inter-observer variability and improving treatment consistency. The module's ability to integrate information across axial, coronal, and sagittal planes ensures that the model's attention mechanisms align with human visual reasoning, enhancing clinical interpretability and trust.

Collectively, the DHDC and CDRSEA modules form a synergistic optimization framework that delivers a reliable, objective, and reproducible tool for glioma subregion segmentation. By combining adaptive scale modeling with cross-dimensional context enhancement, DPEA-Net provides a principled solution that bridges the gap between advanced deep learning techniques and the practical demands of neuro-oncological assessment.

### 4.2 Practical utility as a neuroimaging research tool

The exceptional parameter efficiency of DPEA-Net translates into several key advantages for integration into neuroimaging research workflows:

- **High computational efficiency:** on a standard NVIDIA Tesla P100 GPU, DPEA-Net processes a full MRI volume in under 2 seconds. This fast inference speed supports near-real-time application and facilitates interactive quality checks or integration into high-throughput pipelines.
- **Low deployment barrier:** the model's modest memory footprint allows it to run on ordinary laboratory workstations without requiring high-performance computing clusters, democratizing access to advanced segmentation capabilities.
- **Simplified integration:** the compact model size and standardized input-output format make DPEA-Net straightforward to embed within popular neuroimaging frameworks, promoting adoption for automated preprocessing in large-scale, multi-site neuro-oncology studies. The standardized input format and fast inference make DPEA-Net compatible with existing PACS and radiotherapy planning systems, facilitating seamless integration.

Beyond these technical advantages, DPEA-Net directly supports several high-impact clinical and translational workflows:

- **Radiotherapy target delineation:** leveraging the model's precise segmentation of the TC region (mean Dice >85%), with an average inference time of 1.23 seconds per volume on standard clinical hardware, DPEA-Net generates high-quality initial segmentations almost instantaneously. This rapid turnaround can substantially streamline the radiotherapy planning workflow by providing a consistent and accurate starting point that requires only minimal manual refinement, thereby reducing the overall time burden associated with fully manual contouring. By minimizing inter-observer variability, it provides a consistent, objective reference for clinical target volume definition, supporting adaptive radiotherapy workflows.
- **Intraoperative surgical guidance:** with sub-2-second inference on standard clinical GPUs, DPEA-Net can be integrated into intraoperative MRI systems to provide real-time, high-resolution visualization of tumor boundaries and functional tissue preservation. This capability assists neurosurgeons in maximizing the extent of resection while minimizing damage to critical brain regions.
- **Large-scale epidemiological studies:** its low computational footprint makes DPEA-Net ideally suited for batch processing of thousands of patient scans in multi-center cohort studies. It enables population-level analyses of glioma incidence, radiomic feature correlation, and treatment response, thereby accelerating evidence-based neuro-oncology research.
- **Longitudinal tumor monitoring:** the model's consistent performance across serial MRI scans makes it suitable for objective longitudinal monitoring. It allows clinicians to quantitatively assess tumor growth, regression, or treatment response over time without the confounding effects of manual re-contouring, thereby providing a reliable biomarker for therapeutic evaluation.
- **Multi-center clinical trials:** in neuro-oncology clinical trials, consistent and objective segmentation is essential for imaging-based response assessment criteria (e.g., RANO). DPEA-Net's high reproducibility across scans makes it suitable for multi-center studies where inter-reader variability often confounds treatment effect evaluation.

These features, grounded in rigorous ablation validation, establish DPEA-Net not only as an accurate segmentation model but also as a practical, translatable tool applicable to diverse clinical and research scenarios.

### 4.3 Limitations and future directions

While DPEA-Net achieves competitive performance on standardized BraTS benchmarks and shows clear utility for radiotherapy planning and large-cohort analysis, several limitations remain and point to meaningful future research directions.

First, the current validation is restricted to the curated BraTS 2019/2020 datasets. Although BraTS aggregates multi-institutional data, all images have been co-registered, resampled to isotropic resolution, and expertly annotated, resulting in a level of consistency rarely encountered in raw clinical data. Consequently, DPEA-Net has not yet been evaluated on authentic clinical datasets that present challenges such as variable field strengths, differing scanner manufacturers, heterogeneous acquisition protocols, and common image artifacts. We fully acknowledge that such validation is essential to establish true generalizability. In future work, we plan to address this by partnering with clinical centers to prospectively acquire and test on real-world MRI data, and by participating in ongoing community challenges that focus on domain generalization and domain adaptation for brain tumor segmentation.

Second, while DPEA-Net yields state-of-the-art performance across all glioma subregions, segmentation accuracy for the small-volume ET region remains relatively lower than for WT and TC. This reflects the inherent challenge of segmenting tiny, irregularly shaped lesions with ambiguous boundaries. Future improvements could incorporate clinical prior knowledge or adopt task-specific weighted loss to further boost ET performance.

Third, the current pipeline relies on preprocessed, intensity-normalized MRI inputs as required by the BraTS protocol. While this ensures consistency in experimental evaluation, it introduces dependency on specialized preprocessing workflows that may hinder direct clinical deployment. Future work will aim to develop an end-to-end pipeline integrating DICOM-to-analysis conversion and adaptive intensity normalization, further bridging the gap between research algorithms and clinical practice.

Finally, although DPEA-Net's segmentation accuracy is well-validated, its direct impact on downstream clinical and research tasks remains unexplored. Future directions such as multi-center validation and model interpretability have been highlighted as critical for clinical translation (14). Future studies will link DPEA-Net's outputs to clinical endpoints to fully establish its utility in comprehensive neuro-oncology analysis pipelines.

Addressing these limitations will be critical to transitioning DPEA-Net from a validated research method into a robust, clinically actionable tool widely adoptable by the neuroimaging community.

## 5 Conclusion

In this study, we proposed DPEA-Net, a lightweight 3D convolutional neural network designed for accurate and efficient glioma subregion segmentation from Multiparametric MRI. The model directly addresses three key barriers that limit the clinical adoption of existing 3D CNNs: excessive computational overhead, poor adaptability to tumor heterogeneity, and suboptimal performance on clinically critical small-volume regions.

The two core modules, DHDC and CDRSEA, were systematically validated through ablation and sensitivity analyses. The DHDC unit enables adaptive multi-scale feature extraction with a 99% parameter reduction compared to 3D U-Net, effectively capturing the heterogeneous appearance of gliomas across different patients. The CDRSEA module explicitly models cross-dimensional spatial interactions, mimicking radiologists' MPR review process to refine ambiguous tumor boundaries, a persistent challenge in volumetric neuroimaging analysis. Furthermore, the 1.5 × *TC* weighting strategy significantly enhances segmentation of this clinically critical region without compromising overall performance.

Extensive experiments on the BraTS 2019 and 2020 benchmarks demonstrated that DPEA-Net achieves state-of-the-art segmentation performance, with Dice scores of 90.43%/89.96% (WT), 85.56%/86.52% (TC), and 81.89%/80.31% (ET), while requiring only 17.48 GFLOPs. This exceptional computational efficiency enables sub-2-second inference on standard clinical workstations, overcoming deployment barriers in resource-constrained environments. Qualitative analysis confirmed the model's superior capability in distinguishing tumor infiltration from vasogenic edema, a key clinical challenge in glioma assessment.

The clinical relevance of TC and ET segmentation extends beyond diagnosis. In radiation oncology, the TC guides boost dose delivery, while the ET defines the gross tumor volume (GTV) for treatment planning. In neurosurgical planning, the ET informs biopsy targeting and resection margins. By delineating these regions with high precision and reproducibility, DPEA-Net has the potential to standardize neuro-oncological workflows, reduce inter-observer variability in multi-center trials, and support adaptive radiotherapy and intraoperative guidance.

In summary, DPEA-Net bridges the gap between advanced deep learning techniques and clinical neuro-oncology practice, providing a reliable, efficient, and clinically actionable solution for glioma segmentation. Future work will focus on multi-center validation, extension to other brain tumor types, and the development of an end-to-end pipeline integrating DICOM-to-analysis conversion, further enhancing its translational potential for widespread adoption in the neuroimaging community.

## Data Availability

The raw data supporting the conclusions of this article will be made available by the authors, without undue reservation.
