# Supplementary material for: DPEA-Net: a clinically-oriented lightweight 3D CNN for glioma segmentation in multiparametric MRI
Source: Front Neurol. 2026 May 25;17:1832698. doi: 10.3389/fneur.2026.1832698 (PMC13244002; doi:10.3389/fneur.2026.1832698)
Supplement: Supplementary file 1 [file Data_Sheet_1.pdf]

Supplementary Material

1 DETAILED METHOD

The open-source code and pre-trained weights of DPEA-Net are publicly accessible at https://github.com/jingxuerong/DPEA-Net for full experimental reproducibility.

1.1 Training Convergence Analysis

Figure S1 illustrates the training and validation loss curves as well as the DSC trajectories for the three glioma subregions over 500 epochs. The validation loss plateaus after approximately 250 epochs, with no significant divergence between training and validation curves, indicating the absence of overfitting.

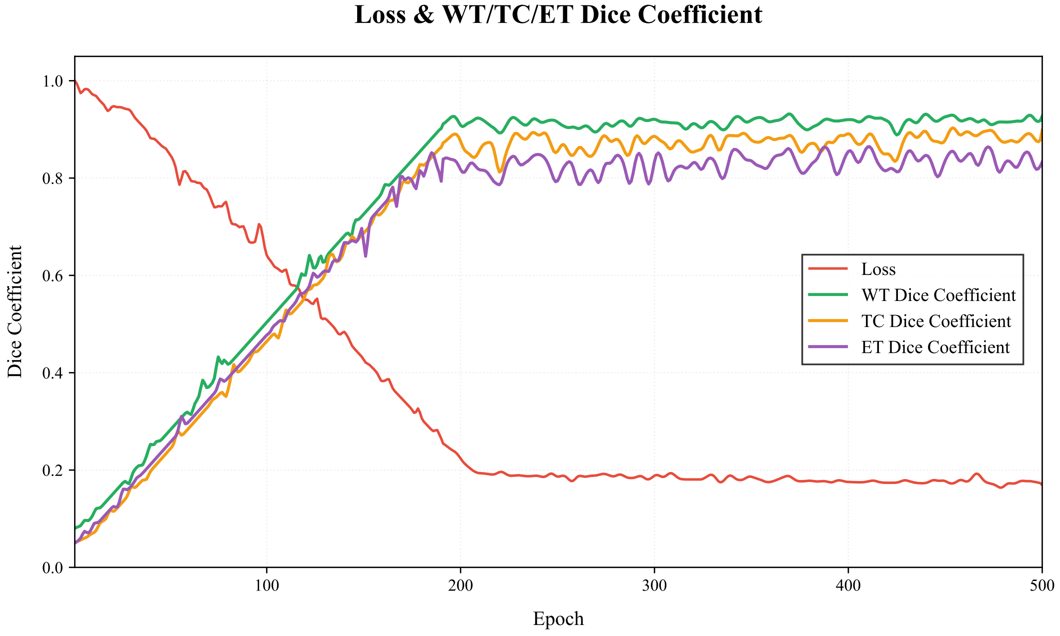

Figure S1. Training dynamics of DPEA-Net on the BraTS 2019 dataset. (a) Generalized Dice Loss (GDL) over epochs; (b)Dice scores for WT, TC, and ET subregions.

1.2 Full Calculation Formulas of the DHDC Unit

The core mathematical formulation of the DHDC unit, including the dynamic partial convolution and the final fusion operation, has been presented in the main text (Section 2.3). For completeness and to facilitate exact reproduction, we provide below the full step-by-step derivation with all intermediate variables explicitly defined.

Given an input feature tensor $X \in \mathbb{R}^{B\times C_{\text{in}}\times D\times H\times W}$, the complete computational pipeline of the DHDC unit is detailed as follows:

1. **Channel projection and activation:**

$$X_1 = \sigma\left(\text{BN}\left(\text{Conv}_{1\times1\times1}(X)\right)\right) \tag{S1}$$

where σ denotes the ReLU activation function, and $X_1 \in \mathbb{R}^{B\times C_{\text{out}}\times D\times H\times W}$ (C_{out} is the number of output channels).

2. **Channel-wise splitting and cascaded dynamic partial convolution:** The tensor X_1 is evenly partitioned along the channel dimension into four sub-tensors $X_1^1, X_1^2, X_1^3, X_1^4$. The latter three branches are processed sequentially with cumulative connections:

$$\begin{cases} X_1^{2'} = \text{DynamicPartialConv}(X_1^2) \\ X_1^{3'} = \text{DynamicPartialConv}(X_1^3 + X_1^{2'}) \\ X_1^{4'} = \text{DynamicPartialConv}(X_1^4 + X_1^{3'}) \end{cases} \tag{S2}$$

where the $\text{DynamicPartialConv}$ operator encapsulates multi-branch dilated convolution with data-driven attention weighting, as defined in Equation 2 of the main manuscript. For reference, its expanded form is:

$$\text{DynamicPartialConv}(Z) = \sigma\left(\text{BN}\left(\text{Conv}_{1\times1\times1}\left(\sum_{k=1}^{3} \alpha_k \cdot B_k(Z) + Z\right)\right)\right) \tag{S3}$$

with $B_k(Z)$ denoting the output of the k-th dilated convolution branch (dilation rates $\gamma \in \{2, 4, 8\}$) and $\alpha_k = \text{Softmax}(\text{Conv}_{1\times1\times1}(\text{GAP}(Z)))$.

3. **Feature concatenation:**

$$X_{\text{fusion}} = X_1^1 \oplus X_1^{2'} \oplus X_1^{3'} \oplus X_1^{4'} \tag{S4}$$

where $\oplus$ denotes channel-wise concatenation.

4. **Residual projection and output:**

$$\hat{X} = \begin{cases} \sigma\left(\text{BN}\left(\text{Conv}_{1\times1\times1}(X)\right)\right), & C_{\text{in}} > C_{\text{out}} \\ X, & C_{\text{in}} \leq C_{\text{out}} \end{cases} \tag{S5}$$

$$Y_{\text{out}} = \sigma\Big(\text{BN}\Big(\text{Conv}_{1\times1\times1}\Big(\hat{X} + X_{\text{fusion}}\Big)\Big)\Big) \tag{S6}$$

where $Y_{\text{out}} \in \mathbb{R}^{B\times C_{\text{out}}\times D\times H\times W}$ is the final output of the DHDC unit.

1.3 Full Calculation Formulas of the CDRSEA Module

The essential mathematical description of the CDRSEA module, including the cross-dimensional attention and region-specific gating mechanisms, is provided in the main text (Section 2.4). The following exposition complements that description with a complete, self-contained, four-stage pipeline definition.

Given an input feature map $X \in \mathbb{R}^{B\times C\times D\times H\times W}$, the CDRSEA module proceeds through the following stages:

1. **Global Spatial Calibration:**

$$\mathcal{A}_{\text{base}} = \sigma\big(\mathcal{A}_{\text{global}}(X_{aHWD})\big) + \sigma\big(\mathcal{M}_{\text{global}}(X_{mHWD})\big) \tag{S7}$$

$$X_{\text{base\_atten}} = \text{Conv}_{1\times1\times1}(X \odot \mathcal{A}_{\text{base}}) \tag{S8}$$

where $\mathcal{A}_{\text{global}}$ and $\mathcal{M}_{\text{global}}$ denote global average pooling and global max pooling, respectively; X_{aHWD} and X_{mHWD} are the tensors obtained after dimension permutation to enable cross-dimensional interaction; $\odot$ denotes element-wise multiplication; and $\mathrm{Conv}_{1\times1\times1}$ represents a $1 \times 1 \times 1$ convolution for adaptive feature projection.

2. **Cross-Dimensional Spatial Enhancement:**

$$X_{\text{cross\_enhanced}} = X_{\text{base\_atten}} + \mathrm{CrossAttn}(X_{\text{base\_atten}}) \tag{S9}$$

where $\mathrm{CrossAttn}(\cdot)$ employs grouped convolutions with anisotropic kernels $(3, 3, 1)$, $(3, 1, 3)$, and $(1, 3, 3)$ to capture planar relationships in the axial, coronal, and sagittal orientations, respectively. The outputs are concatenated and processed by a $1 \times 1 \times 1$ convolution followed by batch normalization and ReLU activation, as detailed in Equation 5 of the main manuscript.

3. **Region-Specific Weighting:**

$$X_{\text{region\_enhanced}} = X_{\text{cross\_enhanced}} \odot \big(1 + \mathrm{RegionAttn}(X_{\text{cross\_enhanced}})\big) \tag{S10}$$

Here $\mathrm{RegionAttn}(\cdot)$ generates three spatial gating maps $g_{\text{WT}}, g_{\text{TC}}, g_{\text{ET}}$ via three independent $1 \times 1 \times 1$ convolutional heads. To mitigate class imbalance, the tumor core (TC) gating coefficient is scaled by a factor of 1.5 prior to the final weighting: $g'_{\text{TC}} = 1.5 \times g_{\text{TC}}$.

4. **Adaptive Gated Fusion:**

$$G = \sigma\big(\mathrm{Conv}_{1\times1\times1}\big(\mathrm{Concat}\big(X \odot \mathcal{A}_{\text{base}},\ X_{\text{region\_enhanced}}\big)\big)\big) \tag{S11}$$

$$Y_{\text{out}} = G \odot (X \odot \mathcal{A}_{\text{base}}) + (1 - G) \odot X_{\text{region\_enhanced}} \tag{S12}$$

where σ denotes the sigmoid activation function, Concat indicates channel-wise concatenation, and Y_{out} is the final output tensor of the CDRSEA module.

1.4 Algorithmic Pseudo-Code of DPEA-Net

To facilitate reproducibility and provide a clear overview of the end-to-end workflow, we present the pseudo-code for training and inference with DPEA-Net in Algorithm 1. The procedure consists of four main stages: (1) data preprocessing, including intensity normalization and hierarchical dimension concatenation (HDC) encoding; (2) model training using the Adam optimizer and Generalized Dice Loss with class weights inversely proportional to squared region volumes; (3) periodic validation and checkpoint selection based on the highest average Dice score on the validation set; and (4) final inference on the test dataset using the best saved model parameters. The network forward pass integrates the DHDC and CDRSEA modules as described in the main manuscript.

1.5 Statistical Significance Testing

Table S1 presents the complete results of paired t-tests comparing DPEA-Net against DFM-Net, HDC-Net, and 3D U-Net on the BraTS 2019 validation set. Tests were conducted for both DSC and HD_{95} across the three glioma subregions (WT, TC, ET). Significance levels are denoted as * $p < 0.05$, ** $p < 0.01$, and *** $p < 0.001$.

Algorithm 1 Training and inference procedure of DPEA-Net

Input: X_{train}, training dataset (multi-parametric MRI volumes).
Input: Y_{train}, ground truth segmentation masks.
Input: X_{val}, validation dataset.
Output: Y_{pred}, predicted segmentation masks for validation/test data.
Output: θ^*, optimized network parameters.

begin
// Step 1: Data Preprocessing
  $X_{\text{train}} \leftarrow \text{IntensityNormalize}(X_{\text{train}})$ {Scale to [0,1]}
  $X_{\text{train}} \leftarrow \text{HDC\_SpatialEncoding}(X_{\text{train}})$ {Hierarchical dimension concatenation}
  $Y_{\text{train}} \leftarrow \text{LabelMapping}(Y_{\text{train}})$ {Convert to 4-class format}
// Step 2: Model Initialization
  Initialize network parameters θ with Kaiming initialization
  optimizer $\leftarrow \text{Adam}(\theta, \text{lr} = 1 \times 10^{-4})$
  best_dice $\leftarrow 0$, $\theta^* \leftarrow \theta$
// Step 3: Training Loop
for epoch = 1 to N_{epochs} **do**
  for batch = 1 to N_{batches} **do**
    $X_b, Y_b \leftarrow \text{GetBatch}(X_{\text{train}}, Y_{\text{train}}, \text{batch\_size})$
    $\hat{Y}_b \leftarrow \text{DPEA-Net}(X_b; \theta)$ {Forward pass through encoder-decoder}
    $\mathcal{L} \leftarrow \text{GDL}(\hat{Y}_b, Y_b)$ {Weights $\propto 1/\text{volume}^2$}
    $\nabla_\theta \mathcal{L} \leftarrow \text{Backward}(\mathcal{L})$
    $\theta \leftarrow \text{optimizer.step}(\theta, \nabla_\theta \mathcal{L})$
  end for
  // Step 4: Validation and Checkpointing
  if epoch $\bmod$ validation_interval $== 0$ **then**
    $\hat{Y}_{\text{val}} \leftarrow \text{DPEA-Net}(X_{\text{val}}; \theta)$
    current_dice $\leftarrow \text{DiceScore}(\hat{Y}_{\text{val}}, Y_{\text{val\_gt}})$
    if current_dice $>$ best_dice **then**
      $\theta^* \leftarrow \theta$
      best_dice $\leftarrow$ current_dice
    end if
  end if
end for
// Step 5: Final Inference
$Y_{\text{pred}} \leftarrow \text{DPEA-Net}(X_{\text{test}}; \theta^*)$ {Apply Test-Time Augmentation if specified}
return $\theta^*, Y_{\text{pred}}$
end

Table S1. Statistical Comparison of DPEA-Net against Baseline Methods

Comparison	Metric	DPEA-Net (A)		Baseline (B)		t-value	p-value	Sig.
		Mean	Std	Mean	Std			
DPEA-Net vs DFM-Net	WT_DSC	0.9043	0.0471	0.8893	0.0425	2.6215	0.0097	**
	WT_HD$_{95}$	4.8700	2.5014	5.3600	2.2100	-2.1350	0.0346	*
	TC_DSC	0.8556	0.0953	0.8379	0.0620	2.4503	0.0161	*
	TC_HD$_{95}$	5.5200	3.2414	6.9228	3.2874	-2.9790	0.0032	**
	ET_DSC	0.8189	0.1550	0.7636	0.1769	4.0190	0.0002	***
	ET_HD$_{95}$	3.0800	2.3717	4.9100	4.9993	-3.6984	0.0004	***
DPEA-Net vs HDC-Net	WT_DSC	0.9043	0.0471	0.8766	0.0967	2.4678	0.0162	*
	WT_HD$_{95}$	4.8700	2.5014	5.7300	2.3700	-2.6850	0.0078	**
	TC_DSC	0.8556	0.0953	0.8127	0.1486	3.3493	0.0013	**
	TC_HD$_{95}$	5.5200	3.2414	8.3889	8.2065	-2.7669	0.0073	**
	ET_DSC	0.8189	0.1550	0.7869	0.1855	2.4820	0.0143	*
	ET_HD$_{95}$	3.0897	2.3888	4.5400	5.5718	-2.4282	0.0180	*
DPEA-Net vs 3D U-Net	WT_DSC	0.9043	0.0471	0.8506	0.1355	3.2399	0.0019	**
	WT_HD$_{95}$	4.8700	2.5014	15.3200	18.2996	-4.7235	¡0.0001	***
	TC_DSC	0.8556	0.0953	0.7996	0.1893	3.1004	0.0028	**
	TC_HD$_{95}$	5.5200	3.2414	15.9951	18.8518	-4.4775	¡0.0001	***
	ET_DSC	0.8189	0.1550	0.7341	0.2735	2.8707	0.0055	**
	ET_HD$_{95}$	3.0800	2.3717	14.0300	20.2322	-4.7354	¡0.0001	***

Abbreviations: WT = Whole Tumor; TC = Tumor Core; ET = Enhancing Tumor; DSC = Dice Similarity Coefficient; HD$_{95}$ = 95th Percentile Hausdorff Distance; Std = Standard Deviation; Sig. = Significance.
Significance levels: * $p < 0.05$, ** $p < 0.01$, *** $p < 0.001$.
